# Effects of Reoperation Timing on Survival among Recurrent Glioblastoma Patients: A Retrospective Multicentric Descriptive Study

**DOI:** 10.3390/cancers15092530

**Published:** 2023-04-28

**Authors:** Ondrej Kalita, Tomas Kazda, Stefan Reguli, Radim Jancalek, Pavel Fadrus, Marek Slachta, Petr Pospisil, Lukas Krska, Jana Vrbkova, Lumir Hrabalek, Martin Smrcka, Radim Lipina

**Affiliations:** 1 Department of Neurosurgery, Faculty of Medicine and Dentistry, Palacky University in Olomouc, University Hospital Olomouc, Zdravotníků 248/7, 779 00 Olomouc, Czech Republic; 2 Department of Health Care Science, Faculty of Humanities, T. Bata University in Zlin, Stefanikova 5670, 760 01 Zlín, Czech Republic; 3 Department of Radiation Oncology, Faculty of Medicine, Masaryk University, Masaryk Memorial Cancer Institute, Zluty Kopec 7, 656 53 Brno, Czech Republic; 4 Department of Neurosurgery, Faculty of Medicine, University of Ostrava, University Hospital Ostrava, 17. Listopadu 1790/5, 708 52 Ostrava, Czech Republic; 5 Department of Neurosurgery, Faculty of Medicine, Masaryk University, St. Anne’s University Hospital in Brno, Pekarska 664/53, 602 00 Brno, Czech Republic; 6 Department of Neurosurgery, Faculty of Medicine, Masaryk University, University Hospital Brno, Jihlavská 20, 625 00 Brno, Czech Republic; 7 Institute of Molecular and Translate Medicine, Faculty of Medicine and Dentistry, Palacky University in Olomouc, Hnevotinska 133/5, 779 00 Olomouc, Czech Republic

**Keywords:** glioblastoma, reoperation timing, treatment strategy

## Abstract

**Simple Summary:**

Given the lack of effective second-line oncotherapy and clear recommendations for surgery for regrowing tumors, the optimal treatment strategy for recurrent glioblastoma (GBM) remains controversial. We strove to identify patients with GBM who achieved survival benefits from repeated surgery, based on reoperation time. A positive but insignificant effects on postsurgical survival were found in univariate analyses for surgery that took place 6 to 16 months after diagnosis. In multivariate analyses, surgical efficacy was insignificant in the range of 6 to 24 months. Significant efficacy was proven for surgery in the 16th month after diagnosis in univariate analyses and in the 22nd month in multivariate analyses. Based on our results, the best outcomes yielded GBM reoperation in the following period after the 16th month of progression, and its efficacy increased up to the 22th month. Reoperations performed less than 6 months after diagnosis, when the tumors had progressed following oncotherapy, had no impact on survival.

**Abstract:**

Glioblastoma inevitably recurs, but no standard regimen has been established for treating this recurrent disease. Several reports claim that reoperative surgery can improve survival, but the effects of reoperation timing on survival have rarely been investigated. We, therefore, evaluated the relationship between reoperation timing and survival in recurrent GBM. A consecutive cohort of unselected patients (real-world data) from three neuro-oncology cancer centers was analyzed (a total of 109 patients). All patients underwent initial maximal safe resection followed by treatment according to the Stupp protocol. Those meeting the following criteria during progression were indicated for reoperation and were further analyzed in this study: (1) The tumor volume increased by >20–30% or a tumor was rediscovered after radiological disappearance; (2) The patient’s clinical status was satisfactory (KS ≥ 70% and PS WHO ≤ gr. 2); (3) The tumor was localized without multifocality; (4) The minimum expected tumor volume reduction was above 80%. A univariate Cox regression analysis of postsurgical survival (PSS) revealed a statistically significant effect of reoperation on PSS from a threshold of 16 months after the first surgery. Cox regression models that stratified the Karnofsky score with age adjustment confirmed a statistically significant improvement in PSS for time-to-progression (TTP) thresholds of 22 and 24 months. The patient groups exhibiting the first recurrence at 22 and 24 months had better survival rates than those exhibiting earlier recurrences. For the 22-month group, the HR was 0.5 with a 95% CI of (0.27, 0.96) and a *p*-value of 0.036. For the 24-month group, the HR was 0.5 with a 95% CI of (0.25, 0.96) and a *p*-value of 0.039. Patients with the longest survival were also the best candidates for repeated surgery. Later recurrence of glioblastoma was associated with higher survival rates after reoperation.

## 1. Introduction

Glioblastoma (GBM) is the most common malignant brain tumor in adults and has a dismal prognosis with near-inevitable recurrence [1]. Aggressive multimodal therapy involving maximally radical and safe tumor resection followed by the Stupp oncotherapy protocol has yielded the best treatment outcomes [2,3,4]. Preoperative and intraoperative imaging tools such as MRI, Raman spectroscopy, fluorescence-guided surgery, and intraoperative brain mapping have made it possible to maximize tumor cytoreduction and minimize surgical morbidity during neurosurgery, significantly improving GBM patients’ life expectancy [5]. However, the rate of increase in patient survival was slow at the beginning of the temozolomide era and has plateaued in the last 5–10 years [6,7].

In the absence of effective second-line oncotherapy or clear recommendations for surgical treatment of regrowing tumors, there is no consensus as to what treatment strategy for recurrent GBM is most effective. Reoperation and reirradiation are commonly used in locoregional therapy. Other common strategies include systemic therapy with alkylating agents (temozolomide, nitrosoureas) and/or antiangiogenic agents (bevacizumab) [8], as well as unclassified TTF therapy [9,10,11]. One of the few known controllable prognostic factors for GBM patients is maximal safe resection [12,13,14,15]. 

Given the lack of effective first- and second-line GBM treatments, reoperation has become a standard medical option for managing this condition. The aim of this retrospective multicentric study was to clarify the conditions under which repeated surgery improves overall survival among GBM patients in order to reduce the incidence of ineffective surgical overtreatment of recurrent GBM. To this end, we analyzed the effects of reoperation timing, clinical condition, and surgical radicality on overall survival among GBM patients. The objective was to identify the subcohort of patients (defined by timing of reoperation) who benefit most from surgery for GBM recurrency or who actually do not have any meaningful benefit from reoperation even if they meet the usual indication criteria. Real-world-data analyses of surgery are an important supplement to clinical and translational research and may be a source of importance, especially for evaluation of timing of second surgeries.

## 2. Materials and Methods

### 2.1. Patients

Data of all glioma patients treated at three Czech neuro-oncology centers (apart from these three centers in the Moravian region (about 4.5 million inhabitants) of Czechia, there are an additional five centers in the Bohemian region) are collected regularly and prospectively during routine maintenance of a national database of operated patients. This work focuses on adult supratentorial recurrent GBM patients who underwent resection and oncotherapy between January 2008 and December 2019. Information on the patients’ clinical conditions (Karnofsky score (KS) and WHO performance status (PS WHO)) from this database was collected along with imaging and histological data on each patient’s tumor and details of their cytogenic alterations. Clinicians at all three centers strove to perform maximal safe and radical primoresection followed by standard aggressive oncotherapy (Stupp protocol) in all GBM patients. All patients underwent early postsurgical MRI (within 72 h) to determine resection radicality. The degree of removal of the original tumor volume was recorded as gross total resection (GTR), subtotal resection (STR), or partial resection [13,16]. All of the tumors were classified by local neuropathologists. The WHO 2007 classifications initially used in the clinical setting were converted into WHO 2016 GBM IDH status classifications in accordance with current recommendations [17,18]. Following diagnosis, patients received periodic checkups with MRI every 3 months until death. Tumor tissue samples were collected in both formalin-fixed, paraffin-embedded (FFPE) forms and fresh-frozen forms. All patients signed informed consent forms for anonymized post hoc analysis of regularly collected data, and this study was approved by local ethics committees (No.2022/1300/MOU). The multidisciplinary tumor board evaluated all patients with tumor progression and considered surgery for recurrent GBM if the following criteria were satisfied and those with histologically proven tumor recurrence were included in this study:

1. The tumor volume increased by >20–30% or a tumor was rediscovered after radiological disappearance.
2. The patient’s clinical status was satisfactory (KS ≥ 70% and PS WHO ≤ gr. 2).
3. The tumor was localized, without multifocality.
4. The minimum expected tumor volume reduction was above 80%.

### 2.2. Investigation of IDH Mutation and MGMT Promotor Methylation

Isocitrate dehydrogenase (IDH) mutation and O6-methylguanine-DNA methyltransferase (MGMT) promoter methylation status were assessed using standard techniques employed at the three neuro-oncology centers described in detail in previous reports [19,20,21]. Briefly, immunohistochemistry (anti-IDH1R132H) and genotyping with Next-Generation Sequencing (Nextera XT kit, Illumina, San Diego, CA, USA) were employed for IDH mutation analyses and real-time methylation-specific PCRs or pyrosequencing analysis of MGMT promoter methylation.

### 2.3. Statistical Methods

The variables evaluated included clinical characteristics (age, sex), genetic markers (IDH, MGMT), and three measures of survival: overall survival (OS, i.e., the time from the day of the first surgery to death or the last follow-up), time to progression (TTP, i.e., the time between the initial and repeat surgeries), and postsurgical survival (PSS, i.e., survival after surgery for the first recurrence). Also included were two variables relating to the characteristics of the initial surgery and treatment after the initial surgery (radicality of the initial surgery and treatment using the Stupp protocol after the initial surgery) and two variables characterizing repeat surgery or surgical intervention after the first recurrence (KS when indicated for repeat surgery and use of the Stupp protocol after the first repeat surgery). Two of these variables were categorical, namely surgical radicality (categorized into GTR, STR, and partial resection) and KS (categories: <80; 80–100).

All data processing was carried out using the R statistical software package (www.r-project.org (accessed on 1 November 2022)), version 4.2.2. The effects of basic variables (sex, radicality of initial surgery, treatment after initial surgery, application of the Stupp protocol, IDH mutation status, and MGMT methylation status) on TTP were evaluated using the Wilcoxon two-sample test. The TTP values for the individual patient subgroups (defined based on the categories mentioned above) were summarized as medians and ranges (min–max). The log-rank test was used to evaluate the effects of sex, IDH mutation, MGMT methylation, KS at the time of indication for repeat surgery, radicality of surgery for the first recurrence, and Stupp protocol adherence after repeat surgery in the time from repeat surgery to death or the last follow-up appointment (PSS). Additionally, a Cox regression model was used to evaluate the effects of sex, IDH, MGMT, and treatment. The effect of TTP (treated as a categorical variable with threshold values of 6, 8, 9, 10, 12, 14, 16, 18, 20, 22, and 24 months) on PSS was evaluated using the log-rank test, a univariate Cox regression model, and a Cox regression model stratified according to categorized KSs at the time of indication for repeat surgery, with adjustment for age. Since KS was the only statistically significant factor in the univariate PSS analysis, no multivariate model was formulated for PSS. The results of log-rank tests are presented using *p*-values while those of Cox regressions are reported as point- and confidence-interval HR estimates together with *p*-values for significance tests. Kaplan–Meier estimates for one- and two-year survival rates were also reported for the categorized time to repeat surgery.

## 3. Results

This analysis included 106 patients aged between 24 and 79 years. The median and mean ages of the patients were 55 years and 54 years, respectively. The group contained slightly more men (58/106 = 54.7%) than women (48/106 = 45.3%). The other basic clinical characteristics are summarized in Table 1. 

The duration of follow-up ranged from 4.1 to 158.6 months, with a mean of 28.5 and a median of 20.3 months. The time to progression (TTP) ranged from 0.1 to 75.5 months, with a mean of 14 months and a median of 10.1 months; the first and third quartile values were 6.2 months and 18.8 months, respectively. It was possible to determine IDH mutation and MGMT methylation status in 76 and 51 patients, respectively. The treatments after the initial surgery and after surgery for the first progression (Surgery-1stPD) were categorized in terms of adherence to the Stupp protocol (information on the applied oncotherapy was missing for four patients). The median OS from the initial surgery was 22.5 months with a 95% CI of (19.4, 29.9) based on data for 88 events (representing 83% of the patients included in this analysis). The median PSS was 9.8 months with a 95% CI of (8.5, 12.5). For further details, see Table 2.

There were no statistically significant differences in TTP between patient groups with different basic characteristics (sex and IDH or MGMT status) or differing radicalities of initial surgery. However, treatment after the initial surgery (categorized in terms of Stupp protocol adherence) had a significant effect (Wilcoxon test; *p*-value = 0.040); the median TTPs for the cohorts with and without Stupp-protocol treatment were 10.7 and 5.8 months, respectively. 

**Figure 1:**
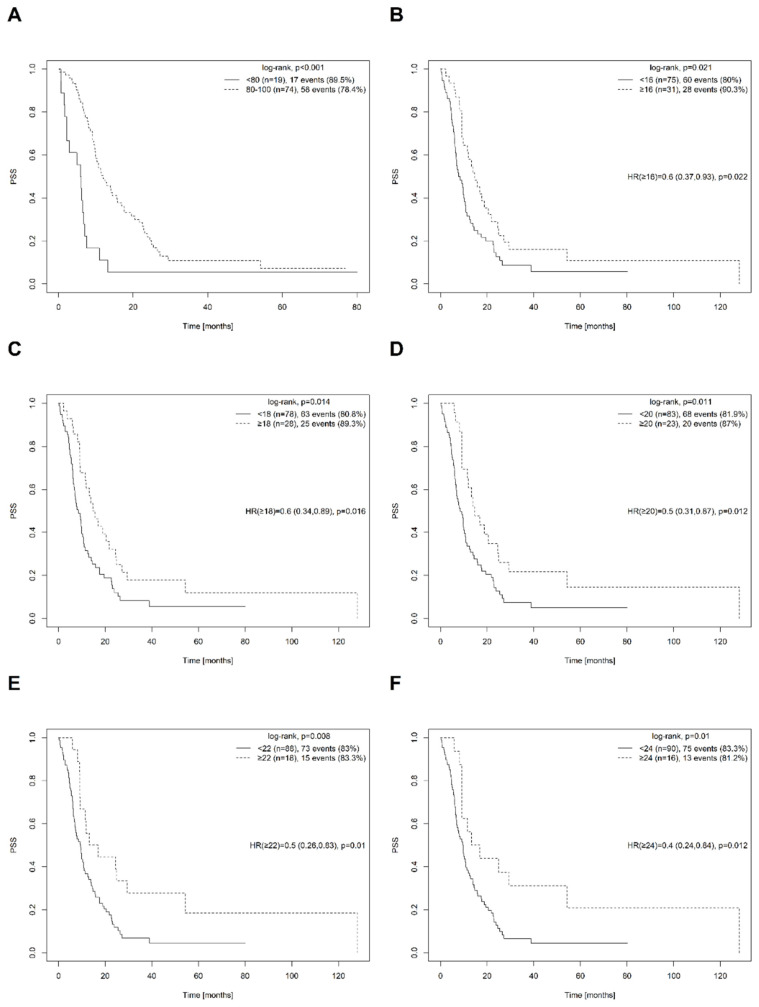
Univariate survival (postsurgical survival-PSS) analysis–estimated Kaplan–Meier survival curves, log-rank test results, and Cox PH model output in the form of point and interval (HR) estimates and significance-test *p*-values (*p*) for the Karnofsky score at the time of surgery for the first progression (Surgery-1stPD) (**A**) and categorized time to progression (TTP) for selected cut-off values (**B**–**F**).

The only variable with a statistically significant effect on PSS, according to the log-rank test, was the categorized KS at the time of Surgery-1stPD (Figure 1A). Radicality of Surgery-1stPD and Stupp-protocol adherence after repeat surgery had no statistically significant influence on PSS (Table 2). It should be noted that only eight patients were treated using the Stupp protocol after Surgery-1stPD; the most common treatment applied to the others was temozolomide chemotherapy alone.

### 3.1. Effect of Progression Time on PSS

All patients in our cohort were operated on after disease progression was detected. We, therefore, investigated the effect of TTP on PSS using univariate modeling. As the interquartile range of TTP was 6.2 to 18.8 months in our cohort, with a maximum of 75.5 months, we tested TTP thresholds of 6, 8, 9, 10, 12, 14, 16, 18, 20, 22, and 24 months. Table 3 shows the results of univariate analyses of survival (PSS) for each TTP threshold, including point HR values with the corresponding 95% confidence intervals and *p*-values from Cox regression models; *p*-values for log-rank univariate tests; and estimated 1-year and 2-year PSS rates for patient subgroups with TTPs above and below the indicated threshold values. The subgroups of patients with TTP values below and above the given threshold values are reported in Table 3. 

Based on the TTP distribution of the patient cohort and the results of the univariate analyses for TTP thresholds of 6–12 months, it cannot be concluded that early progression had a statistically significant effect on PSS. However, significant increases in PSS were observed for patients with relatively late progression, corresponding to TTPs ≥16 months (Figure 1B–F).

### 3.2. Adjusted Model of PPS

The univariate analysis of PPS revealed an effect of the KS at the time of indication for repeat surgery (log-rank test, *p*-value < 0.001), and it is known that both OS and PSS can be influenced by a patient’s age. Multivariate Cox regression models of PSS including these variables were therefore generated. Table 4 shows the output of the CoxPH regression models stratified according to the categorized KSs and adjusted for age for individual TTP threshold values, which shows that the TTP thresholds of 22 and 24 months are associated with statistically significant increases in PSS. Patients operated on for the first recurrence at 22 months (*n* = 18) and 24 months (*n* = 16) had better survival rates than those receiving earlier surgical treatment. For the 22-month group, the HR was 0.5 and the 95% CI was (0.27, 0.96) with a *p*-value of 0.036. For the 24-month group, the HR was 0.5 and the 95% CI was (0.25, 0.96) with a *p*-value of 0.039.

## 4. Discussion

There are only a few controllable factors that are known to affect treatment outcomes among GBM patients, one of which is maximal radical and safe resection. Properly indicated surgery has an important effect on life expectancy and recurrence prognosis. Literature reports have stated that the median survival for GBM patients after a second resection is between 7 and 12.4 months [22,23,24,25]. To identify patients suitable for recurrent GBM surgery, Park et al. developed the NIH Recurrent Glioblastoma Scale 2010, which takes three factors into account: a critical or eloquent tumor location, clinic status, and tumor volume [26]. In 2013, a second scale that incorporated a criterion based on ependymal involvement was introduced [27].

Several exclusion criteria for recurrent GBM resection have also been identified, relating to factors including patient willingness, contraindication or former refusal of oncotherapy following primo-surgery, unfavorable clinical status, partial resection of a primo-surgery, prediction of insufficient surgical radicality, pseudo-progression, and timing of GBM recurrence. This work focuses on the latter of these factors. Indications for repeated surgery in our three neuro-oncology centers included in this retrospective study, as listed in the Materials and Methods section, are pragmatic consensus recommendations reflecting also the ethical point of view and general issues related to real-world practice [28].

Unfortunately, several issues make it impossible to investigate the effect of resection radicality on survival in recurrent GBM patients using a prospective, randomized study. First, the distribution bias of patients makes it difficult to obtain two comparable patient cohorts. Second, tumor features such as morphology, location, and genetic heterogeneity influence both tumor resectability and patient survival, irrespective of surgical interventions. Third, prognostic factors such as age and neurological status influence decision-making concerning aggressiveness of resection, and both factors also influence patient survival independently of surgical intervention. Fourth, patient age, clinical status, and resection radicality all affect decisions concerning oncological therapy during the postoperative period [25]. Finally, it is ethically unacceptable to deliberately retain residuums in young patients with good clinical statuses and whose tumors are favorably located and amenable to radical resection [29,30].

It is commonly accepted that the degree of surgical radicality in prime GBM resections is a positive prognostic factor for PFS and OS, while the presence of a residual tumor is a negative prognostic factor [31,32]. The same principles can be applied to recurrent GBM surgeries by extrapolation [30]. This implies that efficacy of reoperation will depend on radicality of resection and postoperative neurological deficit, exactly as in the primo-surgery [33,34]. Unfortunately, because of the limitations mentioned above, all of the available data on recurrent GBM is derived from nonrandomized clinical trials involving heterogeneous patient cohorts and treatment approaches with diverse endpoints [35]. Despite a general lack of robust evidence, the majority of the available class II and III data support the recommendation of reoperation in recurrent or progressive GBM. The objectives of such surgeries are tumor-burden and peritumoral-edema-mass reduction, corticotherapy dose tapering, stabilization (or even improvement) of neurological status and quality of life, and tumor retyping and/or rephenotyping to provide evidence to support, for example, personalized implementation of off-label oncotherapy recommended by a molecular tumor board. In addition, radicality of resection also influences both the pattern of recurrent disease and repeated tumor resectability. Patients with supratotal resections or GTRs tend to have distant recurrences, while those with partial resections are more likely to suffer local recurrences [36].

Selective pressure imposed by first-line therapies may alter the biological profiles of recurrent tumors. Consequently, a tumor being treated during reoperation may differ markedly from the initial tumor and should be approached as such [37]. In implementing systemic therapy for recurrent GBM, it is common to apply the same cytotoxic agents as were used on the initial tumor, either as monotherapies (temozolomide rechallenge) or in combination with other drugs. However, the results obtained using this approach are not compelling [38,39,40,41,42,43,44,45,46,47,48,49]. Additionally, there have been no positive prospective phase 3 studies supporting efficacy of reirradiation in such cases [50]. Although many different radiotherapeutic techniques have been applied (including stereotactic radiotherapy, intensity-modulated radiotherapy, image-guided radiotherapy, use of hypofractionated schedules, etc.) and despite claims of favorable results in a majority of publications, there is little strong evidence of efficacy [51,52,53,54,55,56,57,58,59,60]. The same appears to be true for efforts to apply tumor-treating-field (TTF) techniques in recurrent GBM [61]. Moreover, no positive effect on life expectancy has been observed for recurrent GBM in trials investigating off-label treatment with pharmaceutical agents and biotechnological interventions including immune checkpoint inhibitors (Nivolumab, Ipilimumab, Pembrolizumab), a PARP inhibitor (Niraparib), adaptive T-cell therapy (CAR-T B7-H3), a topoisomerase inhibitor (Irinotecan), autologous dendritic cells (ADCTA), a FASN inhibitor (ASC40), a PI3K/mTOR inhibitor (Paxalisib), a VEGFR2-TIE2 tyrosine kinase inhibitor (Regorafenib), a JAK1/3 inhibitor (Tofacitinib), oncolytic viruses, and peptide vaccines [36]. There is thus a clear lack of compelling alternatives to reoperation.

Efficacy of recurrent GBM surgery in terms of life expectancy is affected by many of the confounding factors mentioned above. In particular, the timing of GBM recurrence depends on the radicality of the initial resection and the response of the remnant tumor cells to oncotherapy. Both of these outcomes in turn depend on the genetic profiles of the GBM cells. It seems that certain unfavorable biological features lead to early and rapid GBM regrowth, which is a strongly negative prognostic factor associated with reduced OS and PFS [62,63,64,65,66,67,68,69,70,71]. 

Previous studies have generally not specifically evaluated the effect of reoperation timing on survival or treated reoperation timing as a fixed covariate [23,25,27,72,73,74,75,76,77,78,79,80,81,82,83,84,85,86,87]. A few studies and one meta-analysis did examine time dependence of reoperation [88,89,90,91] but yielded no clear conclusion. Only recently, the work by Clavreul et al. published in 2022 evaluated time to the first recurrence by analyzing patients from French glioblastoma biobanks, also showing better outcomes in patients defined as having long (≥18 months) times after the initial surgery (13.0% of all 338 included patients) [92]. Our cohort validates their observation, although they cannot be completely comparable groups of patients. In our cohort, patients with “secondary glioblastoma” were also enrolled, which was mirrored in a higher median TTP (7.8 vs. 10.1 months) and median OS (19.8 vs. 22.5 months) as well as a higher proportion of patients reoperated on after ≥18 months (25% in our cohort). The concern of time of surgery within recurrent GBM as an unknown factor, which could modify reoperation efficacy, is our contribution to this novel issue. For ethical reasons, only a larger number of such retrospective studies can provide sufficient summary evidence for the generalizability of the conclusions described.

In our study, the median OS for the full patient cohort was 22.5 months. The median PSS was 9.8 months. Univariate analysis of the relationship between the reoperation timing for recurrent GBM and PSS revealed a positive but nonsignificant effect on PSS for surgeries conducted between 6 and 16 months after the first diagnosis. Additionally, multivariate analyses revealed no statistically significant effect of surgery at any point between 6 and 22 months after diagnosis. However, significant efficacy was proven for surgical interventions after 16 months in univariate analyses and after 22 months in multivariate analyses. We also confirmed a positive relationship between OS and clinical conditions. No significant relationship was found between OS, resection radicality, and repeated oncotherapy or any molecular marker.

Our results show that the best outcomes among recurrent GBM patients occurred when the TTP was at least 16 months (or 22 months based on multivariate analysis). Reoperative surgery within 6 months of initial diagnosis, meaning that tumor progression occurred while the patient was receiving oncotherapy, had little impact on survival. The effect of surgery for TTP values between these two extremes was unclear, suggesting that reoperation may be acceptable in this window. Identifying patients who could benefit from such reoperation would require careful analysis of individual cases. 

The TTP and PSS in any given case will depend primarily on the genetic characteristics of the patient’s tumor. Unfavorable genetic tumor characteristics are associated with increased age at GBM presentation and poor underlying clinical conditions. GBM cases with adverse genetic features have shorter TTP and PSS values [27] as well as limited responsiveness to oncotherapy [39] and repeated surgery. Our results suggest that to avoid surgical overtreatment of patients with recurrent GBM, it is important to carefully select patients for reoperative surgery based on the clinical and radiological features of their recurrence and the timing of the surgery relative to the initial diagnosis. However, the results presented herein show that distinguishing early recurrence from pseudo-progression is essential. In our study, radicality of repeated resections had a nonsignificant effect on life expectancy. As surgery for GBM recurrence was suggested in our cohort only if the expected tumor volume reduction were more than 80%, we can speculate about biased efficacy-of-indication criteria. For example, it is possible that patients with IDHmt, according to the old classification of secondary GBM, have more extensive and more infiltrating disease, which can then, unfortunately, no longer be indicated for reoperation. This would also explain that no positive prognostic effect of IDH/MGMT was observed on TTP in our cohort.

We also had to keep in mind that reoperation can prolong survival if resection can be achieved without excessive risk of neurologic deterioration [33]. The dominant second oncotherapy among our cohort was temozolomide rechallenge, but no significant effect of second oncotherapy on PSS was established. Only a few patients received reirradiation, and the effect of this treatment remains unclear. Finally, while it should be noted that we lacked genetic information for some patients, we were unable to corroborate any effect of IDH or MGMT status on PSS. 

## 5. Conclusions

Because of the unfavorable outcomes of GBM therapy, clinicians often perform repeated surgery for want of better options. In accordance with our previous reports, we confirmed that radicality of tumor resection and clinical status are positive prognostic factors for both initial and repeat surgical interventions in GBM patients. The response to oncotherapy and its ability to eradicate microscopic remnant GBM cells in the brain tissue surrounding the postresection cavity depends primarily on a tumor’s genetic profile. However, although the majority of GBMs included in our study lacked IDH mutations, there were cases of prolonged survival within the studied cohort. Positive responses to oncotherapy among these patients appear to have encouraged compliance with the Stupp protocol. The aim of this study was to evaluate the effect of surgical timing on survival in recurrent GBM by evaluating real-world data. Patients with positive responses to oncotherapy had the best clinical conditions and the longest survival and were also the best candidates for repeated surgery. Moreover, later recurrence of GBM in relation to initial diagnosis and surgery was associated with higher survival rates after reoperation. As a result, patients with shorter TTPs appear to have less benefit from repeated surgery, with risk related to neurosurgery performance also taken into account. The majority of patients with prolonged survival underwent repeated temozolomide chemotherapy after reoperation but exhibited limited responsiveness. These results highlight the need for appropriate selection criteria to guide identification of optimal treatment strategies for specific patients as well as the urgent need for effective second-line GBM oncotherapy.

GBM recurrence is a dynamic biological process, which makes it difficult to delineate precise timing thresholds for reoperation. However, the results presented herein suggest that reoperative surgery can have a significant positive impact on survival if performed when a certain minimum amount of time has elapsed since the initial diagnosis. 

## 6. Study Limitations

Determination of MGMT methylation and IDH mutation status were only fully incorporated into the diagnostic process at the three Czech neuro-oncology centers in 2011–2015. Consequently, data on MGMT methylation and IDH mutation were only available for roughly two-thirds and half of the studied cohort, respectively. Despite the prospective nature of our glioma database, there were some missing data. The final limitation is that for ethical reasons, no unoperated control group was available for comparison: for example, patients treated without reoperation. The addition of another cohort, after matching patient characteristics between the cohorts, would allow for more statistical comparisons and more reliable result-supported conclusions. On the other hand, focusing on solely patients indicated for reoperation in the frame of real-world practice may bring valid analysis of the effect of time on second surgeries. A comparison of cohorts undergoing repeated surgery with those who received chemotherapy was only recently performed by Gonzáles et al., showing no effect of repeated surgery on PSS.

## Figures and Tables

**Table 1:** Basic clinical characteristics of patient dataset. TTP, time to progression; CI, confidence interval; SE, standard error; IDH, isocitrate dehydrogenase; wt, wild type; mt, mutation; MGMT, O6-methylguanine-DNA methyltransferase; GTR, gross total resection; STR, subtotal resection; RT, radiotherapy; CHT, chemotherapy.

Variables	Median (Min–Max); Number (%)
**Age**	
Years	55 (24–79)
**Follow-Up**	
Months	20.3 (4.1–158.6)
**Time to Progression (TTP)**	
Months	10.1 (0.1–75.5)
**Overall Survival (OS)**	
Events (%)	88 (83%)
Median (95% CI)	22.5 (19.4, 29.9)
1y-surv. ± SE[%]	85 ± 3.5
2y-surv. ± SE[%]	48 ± 5.1
**Post-Progression Surgery Survival (PSS)**	
Events (%)	88 (83%)
Median (95% CI)	9.8 (8.5, 12.5)
1y-surv. ± SE[%]	40 ± 5
2y-surv. ± SE[%]	33 ± 6
**Sex**	
Women	48 (45.3%)
Men	58 (54.7%)
**IDH Methylation Status**	
wt	67 (88.2%)
mt	9 (11.8%)
Unknown	30 patients
**MGMT Methylation**	
No	30 (58.8%)
Yes	21 (41.2%)
Unknown	55 patients
**Radicality of Initial Surgery**	
GTR	60 (60.6.%)
STR or Partial Resection	39 (39.4%)
Unknown	7 patients
**Stupp Protocol After Initial Surgery**	
No	12 (11.8%)
Yes	90 (88.2%)
Unknown	4 patients
**Oncotherapy After Initial Surgery**	
No therapy	5 (4.9%)
RT Only	7 (6.9%)
ChemoRT + Adj. CHT	71 (69.6%)
ChemoRT Only	14 (13.7%)
RT Only and Then CHT Only	5 (4.9%)
Unknown	4 patients
**Karnofsky Score (Before Surgery-1stPD)**	
<80	19 (20.4%)
80–100	74 (79.6%)
Unknown	13 patients
**Radicality of Surgery-1stPD**	
GTR	40 (56.3%)
STR or Partial	31 (43.7%)
Unknown	35 patients
**Oncotherapy After Surgery-1stPD**	
No Therapy	8 (13.1%)
RT	4 (6.6%)
CHT	40 (65.6%)
ChemoRT and Adj. CHT	4 (6.6%)
ChemoRT	2 (3.3%)
RT Only and Then CHT Only	2 (3.3%)
Other	1 (1.6%)
Unknown	45 patients

**Table 2:** Results of univariate analyses of the correlations between time to progression (TTP) and selected factors based on the Wilcoxon two-sample test, and influence on postsurgical survival (PPS) as determined with the log-rank test and Cox PH univariate models (in cases where the proportional hazard assumption was satisfied).

Characteristic	N	TTP		PSS		
	(Total 109)	Median (Min–Max)	*p*-Value *	Log-Rank, *p*-Value	Cox Regression, HR (95% CI)	Cox Regression, *p*-Value
**Sex**						
Female	48	10.5 (0.1–32)	0.399	0.132		
Male	58	9.8 (1.5–75.5)			1.4 (0.9, 2.14)	0.133
**IDH status**						
Wild-type	67	9.3 (0.1–55.7)	0.436	0.263		
Mutated	9	11.2 (3.9–48.1)			0.6 (0.26, 1.45)	0.267
**MGMT Status**						
Unmetylated	30	8.2 (1.5–28.3)	0.645	0.379		
Metylated	21	8.2 (1.9–25.4)			0.7 (0.38, 1.45)	0.381
**Radicality of Initial Surgery**				-	-	
GTR	60	10.3 (0.6–73.6)	0.652			
STR or Partial Resection	39	9.7 (0.1–75.5)				
**Stupp Protocol After Initial Surgery**				-	-	
No	12	5.8 (0.1–27)	0.040			
Yes	90	10.7 (1.2–75.5)				
**Karnofsky Score Before Surgery-1stPD** ^#^						
<80	19	9.3 (1.9–25.4)	0.394	< 0.001		
80–100	74	10.9 (0.1–75.5)				
**Radicality of Surgery-1stPD** ^#^		-				
GTR	40			0.511		
STR or Partial Resection	31					
**Stupp Protocol After** **Surgery-1stPD**		-				
No	53			0.968		
Yes	8				1 (0.44, 2.21)	0.968

* Wilcoxon two-sample test. ^#^ The Cox regression model could not be used due to violation of the proportional hazard assumption; PH test, *p*-value < 0.05.

**Table 3:** Univariate survival analysis (Kaplan–Meier estimates of median survival with 95% confidence interval, Kaplan–Meier estimates of 1-year and 2-year survival, univariate Cox PH model, log-rank test) of post-progression surgery survival (PSS) for subgroups defined according to the selected time to progression (TTP) threshold values. HR = hazard ratio, pV = *p*-value from Cox model, SE = standard error, N = the number of patients in the subgroup. * statistically significant result.

		N	Median (95% CI)	1y-Surv. ± SE [%]	2y-Surv. ± SE [%]	HR (95% CI)	pV	Log-Rank, *p*-Value
6	<cutoff	26	12.9 (7.7, NA)	63.8 ± 10.4	34.8 ± 11.1			0.131
	≥cutoff	80	9.5 (7.9, 11.9)	91.3 ± 3.2	51.7 ± 5.7	1.5 (0.88, 2.68)	0.134	
8	<cutoff	42	9.7 (6.9, 19.5)	67.9 ± 7.7	26.6 ± 7.5			0.706
	≥cutoff	64	9.9 (8.5, 13.9)	95.3 ± 2.6	60 ± 6.2	1.1 (0.7, 1.7)	0.706	
9	<cutoff	48	9.2 (6.4, 15.9)	70 ± 7	27.5 ± 7			0.987
	≥cutoff	58	9.9 (9.1, 14.4)	96.6 ± 2.4	62.7 ± 6.5	1 (0.65, 1.53)	0.987	
10	<cutoff	53	7.7 (6.1, 12.9)	71.1 ± 6.6	24.4 ± 6.4			0.441
	≥cutoff	53	10.8 (9.1, 15.7)	98.1 ± 1.9	68.7 ± 6.5	0.8 (0.56, 1.29)	0.441	
12	<cutoff	64	7.7 (6.7, 11)	74.7 ± 5.7	22.3 ± 5.6			0.082
	≥cutoff	42	13.2 (9.9, 18.8)	100 ± 0	82.9 ± 5.9	0.7 (0.45, 1.05)	0.083	
14	<cutoff	72	8.5 (6.7, 11)	77.7 ± 5.1	26.4 ± 5.6			0.059
	≥cutoff	34	13.6 (9.9, 20.5)	100 ± 0	88.2 ± 5.5	0.7 (0.42, 1.02)	0.061	
16 *	<cutoff	75	8.5 (6.7, 10.8)	78.7 ± 4.9	26.6 ± 5.5			0.021
	≥cutoff	31	14.7 (9.9, 24.5)	100 ± 0	93.5 ± 4.4	0.6 (0.37, 0.93)	0.022	
18 *	<cutoff	78	8.5 (6.8, 10.7)	79.5 ± 4.7	29.9 ± 5.6			0.014
	≥cutoff	28	15.2 (11.5, 24.9)	100 ± 0	92.9 ± 4.9	0.6 (0.34, 0.89)	0.016	
20 *	<cutoff	83	8.5 (6.8, 10.8)	80.9 ± 4.5	31.8 ± 5.5			0.011
	≥cutoff	23	14.7 (11.5, 29.4)	100 ± 0	100 ± 0	0.5 (0.31, 0.87)	0.012	
22 *	<cutoff	88	9.5 (6.9, 11.3)	82 ± 4.2	36.2 ± 5.4			0.008
	≥cutoff	18	15.1 (9.2, NA)	100 ± 0	100 ± 0	0.5 (0.26, 0.83)	0.010	
24 *	<cutoff	90	9.7 (7, 11.9)	82.4 ± 4.1	37.8 ± 5.4			0.010
	≥cutoff	16	15.1 (9.1, NA)	100 ± 0	100 ± 0	0.4 (0.24, 0.84)	0.012	

**Table 4:** Results of age-adjusted Cox PH models of postsurgical survival with Karnofsky-score stratification for selected time-to-progression thresholds. * statistically significant result.

Factor	HR (95% CI)	*p*-Value
Age	1 (0.99, 1.03)	0.220
TTP ≥ 6	1.4 (0.75, 2.6)	0.295
Age	1 (0.99, 1.03)	0.204
TTP ≥ 8	1.1 (0.68, 1.78)	0.705
Age	1 (0.99, 1.03)	0.210
TTP ≥ 9	1.1 (0.68, 1.74)	0.721
Age	1 (0.99, 1.03)	0.206
TTP ≥ 10	1 (0.62, 1.57)	0.945
Age	1 (0.99, 1.03)	0.208
TTP ≥ 12	0.8 (0.48, 1.27)	0.321
Age	1 (0.99, 1.03)	0.201
TTP ≥ 14	0.8 (0.47, 1.27)	0.308
Age	1 (0.99, 1.03)	0.213
TTP ≥ 16	0.7 (0.41, 1.15)	0.154
Age	1 (0.99, 1.03)	0.212
TTP ≥ 18	0.6 (0.37, 1.07)	0.085
Age	1 (0.99, 1.03)	0.275
TTP ≥ 20	0.6 (0.33, 1.05)	0.071
Age	1 (0.99, 1.03)	0.275
TTP ≥ 22 *	0.5 (0.27, 0.96)	0.036
Age	1 (0.99, 1.03)	0.311
TTP ≥ 24 *	0.5 (0.25, 0.96)	0.039

## Data Availability

Due to privacy and confidentiality, patient data is not available. Part of this data was presented at the European Association of Neuro-Oncology (EANO) annual virtual meeting in October 2022.
